# Epigenetic Regulation of Peroxisome Proliferator-Activated Receptor Gamma Mediates High-Fat Diet-Induced Non-Alcoholic Fatty Liver Disease

**DOI:** 10.3390/cells10061355

**Published:** 2021-05-31

**Authors:** Tahar Hajri, Mohamed Zaiou, Thomas V. Fungwe, Khadija Ouguerram, Samuel Besong

**Affiliations:** 1 Department of Human Ecology, Delaware State University, Dover, DE 1191, USA; sbesong@desu.edu; 2 The Jean-Lamour Institute, UMR 7198 CNRS, University of Lorraine, F-54000 Nancy, France; mohamed.zaiou@univ-lorraine.fr; 3 Department of Nutritional Sciences, School of Nursing and Allied Health Sciences, Howard University, Washington, DC 20059, USA; thomas.fungwe@howard.edu; 4 UMR1280 PhAN, Physiopathology of Nutritional Adaptations, INRA, University of Nantes, CHU Hôtel Dieu, IMAD, CRNH Ouest, 44000 Nantes, France; khadija.ouguerram@univ-nantes.fr

**Keywords:** non-alcoholic fatty liver disease (NAFLD), epigenetic, DNA methylation, high-fat diet

## Abstract

Non-alcoholic fatty liver disease (NAFLD) is highly prevalent in Western countries and has become a serious public health concern. Although Western-style dietary patterns, characterized by a high intake of saturated fat, is considered a risk factor for NAFLD, the molecular mechanisms leading to hepatic fat accumulation are still unclear. In this study, we assessed epigenetic regulation of peroxisome proliferator-activated receptor γ (PPARγ), modifications of gene expression, and lipid uptake in the liver of mice fed a high-fat diet (HFD), and in hepatocyte culture challenged with palmitic acid. Bisulfate pyrosequencing revealed that HFD reduced the level of cytosine methylation in the *pparγ* DNA promoter. This was associated with increased expression of the hepatic PPARγ, very low-density lipoprotein receptor (VLDLR) and cluster differentiating 36 (CD36), and enhanced uptake of fatty acids and very low-density lipoprotein, leading to excess hepatic lipid accumulation. Furthermore, palmitic acid overload engendered comparable modifications in hepatocytes, suggesting that dietary fatty acids contribute to the pathogenesis of NAFLD through epigenetic upregulation of PPARγ and its target genes. The significance of epigenetic regulation was further demonstrated in hepatocytes treated with DNA methylation inhibitor, showing marked upregulation of PPARγ and its target genes, leading to enhanced fatty acid uptake and storage. This study demonstrated that HFD-induction of *pparγ* DNA promoter demethylation increased the expression of PPARγ and its target genes, *vldlr* and *cd36*, leading to excess lipid accumulation, an important initiating mechanism by which HFD increased PPARγ and lipid accumulation. These findings provide strong evidence that modification of the *pparγ* promoter methylation is a crucial mechanism of regulation in NAFLD pathogenesis.

## 1. Introduction

Non-alcoholic fatty liver disease (NAFLD) has become an important public health problem because of its high prevalence; potential progression to severe disease; and association with obesity, insulin resistance, and Type 2 diabetes [1,2]. Some studies have indicated that about 20–30% of the general population in Western countries suffer from NAFLD [1,2,3], and the predictions are on the rise considering the prevalence of obesity in the world [4]. If not treated, NAFLD could induce inflammation in liver cells leading to non-alcoholic steatohepatitis (NASH) and possibly irreversible liver cirrhosis [1,4]. Obesity-associated NAFLD is mostly caused by excess energy intake and increased free fatty acids (FFAs) and triglycerides (TGs) in blood, leading to excessive fat accumulation in the liver [5]. Diet composition is also an important factor because it has been reported that diets containing excessive amounts of saturated fat and sugar, commonly called Western-style diet, are a major contributing factor in NAFLD [6]. In fact, some investigations suggested that macronutrient composition in the diet can impact NAFLD, even without any change in body weight [1].

Epigenetic modifications, defined as the chemical changes in DNA and histones without concomitant alterations in nucleotide sequence, are now progressively recognized as major regulators of gene expression and disease development [7]. DNA methylation is among the most extensively studied epigenetic modifications and occurs at the 5′ position of cytosines, primarily in the context of a CpG dinucleotide sequence, to form 5-methylcytosine. This epigenetic modification, which is catalyzed by DNA methyltransferases (DNMT), alters chromatin structure, often leading to transcriptional inhibition and suppression of gene expression [8]. While the pathophysiology of NAFLD has not been fully elucidated, recent investigations have indicated that this disorder may be linked to several factors, including metabolic, environmental, and genetic determinants [8]. Increasing amounts of evidence indicate that nutrients and diets regulate gene expression through DNA methylation [7,8]. Most importantly, the Western-type diet, which is known to promote obesity, also alters DNA methylation [9], thereby changing the expression of several genes involved in lipid metabolism [10,11,12]. Moreover, several in vitro investigations indicated that DNA methylation patterns induced by dietary fatty acids are specifically linked with dysfunction in cellular lipid metabolism and fatty acid oxidation [8,13,14]. However, it is not known if saturated fatty acids exert specific effects on epigenetic landmarks and DNA methylation that may channel FA towards lipid accumulation in hepatocytes, promoting NAFLD pathogenesis [13]. Understanding the patterns of DNA methylation through the interaction with nutrients is fundamental, not only to gain information about pathophysiological mechanisms for the development and progression of NAFLD, but also to pave the way for possible preventive strategies by modifying food consumption.

Peroxisome proliferator-activated receptor γ (PPARγ) is a nuclear transcription factor highly expressed in adipose tissue, where it plays the crucial function of lipid storage [15]. Activation of PPARγ in adipocytes increases the expression of an array of genes whose protein products mediate TG-rich lipoprotein catabolism, FA uptake, and intracellular lipid storage [16]. Very low density lipoprotein receptor (VLDLR) and cluster differentiating 36 (CD36) are known target genes of PPARγ, whose expression is induced by the specific binding of PPARγ to PPAR response elements (PPREs) sequence in the gene promoter [15,16]. While CD36 is known to facilitate fatty acid uptake [17], VLDLR is a receptor of TG-rich lipoproteins, and both of them contribute to enhanced lipid uptake and storage [15,17]. In contrast to adipose tissue, PPARγ expression in liver is very low, as is that of VLDLR and CD36 [17]. Although hepatic expression of PPARγ is upregulated under certain pathophysiologic conditions, such as diabetes, obesity, and high-fat diet [18,19], the mechanisms leading to this induction are still to be revealed. In addition, it is still unknown whether the modulation of PPARγ expression in hepatocyte is linked to the expression of VLDLR and CD36, thereby controlling the rate of hepatic lipid uptake and accumulation. Accordingly, we have undertaken this study to examine potential epigenetic regulation of hepatic PPARγ expression in mice fed a Western-type high-fat diet. Additionally, we investigated the association between epigenetic regulation, expression of PPARγ and its target genes *vldlr* and *cd36*, and fat accumulation in the liver.

## 2. Methods

### 2.1. Animals and Diets

Wild-type C57BL6 mice were purchased from The Jackson Laboratory (Bar Harbor, ME, USA), and were housed in a facility with a 12-h light cycle. Mice were assigned randomly to two groups (16 per group) and were fed a low-fat diet (LFD) and a high-saturated-fat diet (HFD) for a 12 week-period. In the HFD, fat provided 60% energy, mainly as saturated fat (palm oil) in which palmitic acid was the predominant fatty acid, whereas the LFD diet provided 10% calories from fat and instead contained cornstarch (Supplementary Table S1) [20]. After overnight fast, mice were sacrificed under anesthesia, and blood and organs were collected. All animal procedures were approved by the Institutional Animal Care and Use Committee, performed at Hackensack University Medical Center AAALAC-accredited facilities. All animal investigations were performed according to the Guide for the Care and Use of Laboratory Animals published by the National Institutes of Health.

### 2.2. Biochemical Analysis

Triglycerides (TGs), cholesterol, and free fatty acids (FFAs) were assayed enzymatically with available commercial kits, as described previously [15,21]. Plasma levels of tumor necrosis factor alpha (TNF-α) and interleukin-6 (IL-6) were tested by enzyme-linked immunosorbent assays (ELISA), according to the manufacturer’s recommendation as previously reported [22,23].

### 2.3. Liver Histology

Samples of liver tissues were preserved in 10% buffered formalin solution at pH 7.0, washed with phosphate buffered saline, and embedded in paraffin. Paraffin blocks were sectioned to a thickness of approximately 4–5 µm, mounted on slides, and stained with hematoxylin and eosin (H&E). Sections were viewed under inverted light microscope (Zeiss, White Plains, NY, USA) [22,24].

### 2.4. Cell Culture and Treatments

Hepatocytes were isolated from mice by liver perfusion through the portal vein with a collagenase-protease solution (Vitacytes, Indianapolis, IN, USA) [25]. Isolated and washed hepatocytes were seeded in collagen-coated plates and maintained for a period of 4 h in low-glucose DMEM medium supplemented with 20% horse serum, 5% fetal calf serum, 2 mM L-glutamine, and 1% penicillin/streptomycin at 37 °C in a humidified incubator aerated with 5% CO_2_. Samples of hepatocytes isolated from mice fed LFD and HFD diets were immediately frozen at −80 °C to assess gene and protein levels. Additional cell cultures were used to assess the uptake of FA and VLDL, as described below. Cells isolated from mice fed chow diet were used to examine the effects of palmitic acid on DNA methylation and expression of PPARγ, VLDLR, and CD36. To this end, cells were washed with PBS and incubated in fresh low-glucose DMEM medium containing 1% free fatty acids-bovine serum albumin (BSA) (control cells) or medium supplemented with BSA-bound palmitic acid at 50 or 200 µM final concentrations. BSA-palmitic acid (molar ratio 4:1) complex was prepared as previously described [15,22]. At the end of the 48-h treatment period, cells were washed, harvested, and stored at −80 °C for the analysis of *pparγ* DNA promoter methylation, and for protein and gene expression. Additional sets of hepatocytes isolated from chow diet-fed mice were used to test the effects of methylation inhibitor 5′-aza, 2′ deoxycytidine (5-aza-C) (Sigma–Aldrich, Burlington, CA, USA) on the expression of selected genes. It has been shown that 5-aza-C is a specific inhibitor of DNMTs and could be used at low concentrations without inducing cell toxicity [26]. Hence, cells were challenged with 5-aza-C for 48 h, then harvested and stored at −80 °C to assess the expression of *pparγ*, *cd36*, and *vldlr* using quantitative polymerase chain reaction (qPCR).

### 2.5. Fatty Acid and Very Low-Density Lipoprotein Uptake in Isolated Hepatocytes

Hepatocyte fatty acid uptake was assessed with a QBT fatty acid uptake assay kit (Molecular Probes, Eugene, CA, USA), based on the procedure of Liao et al. [27], which used a cell impermeable Bodipy-FA probe, a fluorescently fatty acid analogue. Hepatocytes (5 × 10^4^/well), cultured in 96-well plates, were serum starved for 4 h. Then, FA uptake was performed in balanced salt solution (HBSS)/20 mM Hepes mixture with the addition of Bodipy-FA mixed with 0.2% BSA. Fluorescence reading was performed with a SpectraMax M5 microplate reader at 485ex/515em with bottom plate read. Total fatty acid uptake was assessed as an endpoint point final reading after one-hour incubation [27]. Additionally, cells were viewed under fluorescence microscope (Zeiss, White Plains, NY, USA) to assess intracellular fluorescence, which represented the amount of FA taken into the cells. To assess the uptake of very low density lipoprotein (VLDL) uptake, VLDL were isolated from the plasma of chow diet-fed mice with ultracentrifugation (d < 1.006 g/mL) and were labeled with DiI (1,1′-dioctadecyl- 3,3,3′,3′-tetramethylindocarbocyanine perchlorate) according to the procedure described in previous studies [15]. DiI is a lipid specific dye used to trace the uptake of VLDL lipids [15]. Labeled VLDLs were added to hepatocyte cultures, and incubations were carried for an additional 4 h. Then, cells were extensively washed with PBS and viewed under fluorescence microscope [15].

### 2.6. Cell Viability Assay

Cell viability was examined for each of the experimental conditions indicated using tetrazolium salt3-(4,5-dimethylthiazol-2-yl)-2,5-diphenyltetrazolium bromide (MTT) (Cayman Chemical, Ann Arbor, MI, USA) and lactate dehydrogenase (LDH) (Biovision, Milpitas, CA, USA) assays, as reported earlier [22].

### 2.7. Global DNA CpG Methylation Analysis

DNA was extracted from liver and hepatocytes with a DNeasy DNA extraction kit (Qiagen, Germantown, MD, USA. Global DNA 5-methylcytosine (5-mC) was quantified using the 5-mC ELISA DNA kit according to the manufacturer’s instruction (Zymo Research, Irvine, CA, USA). The procedure was based on the detection of the 5-methyl CpG across a wide range of starting DNA with specific anti-5-mC monoclonal antibody. The 5-mC percentage was quantified based on a standard curve, generated by combining the positive (100%) and negative (0%) controls. The complex issued from the reaction DNA-antibody was visualized by horseradish peroxidase, and the absorbance at 450 nm was measured using a plate reader.

### 2.8. DNA Methyltransferase Activity

The activity of DNA methyltransferase (DNMT) was performed using a commercial kit from Epigentek (Farmingdale, NY, USA). The procedure was based on the reaction of DNMT in tested samples with a substrate coated in the plate well and reaction of the issued product with an anti−5-methylcytosine antibody. The amount of methylated DNA, which was proportional to enzyme activity, was measured by ELISA reaction and by reading the absorbance at 450 nm. The activity of DNMT enzyme was proportional to the intensity of optical density. Assays were performed in duplicate using 10 µg of nuclear extracts.

### 2.9. Analysis of PparγPromoter-Specific DNA Methylation

The methylation status of the proximal region of *pparγ* was assessed by the pyrosequencing of bisulfate treated DNA, based on the procedure described by Delaney et al. [28]. Highly purified genomic DNA was first treated with a bisulfate kit [28] and was amplified with the PyroMark PCR Kit (Qiagen) starting with 95 °C for 5 min, then 45 cycles of 95 °C for 30 s and 58 °C for 30 s (annealing temperature), followed by 72 °C for 30 sec and 72 °C for 10 min. Complete PCR reaction and product sizes were confirmed with 2% agarose gel electrophoresis. Biotinylated PCR products (10 µL) were mixed with streptavidin-coated Sepharose beads (1 μL) and PyroMark binding buffer (40 μL) in an 80 μLtotal volume reaction. Then, PCR products were purified by running the mixture in a PyroMark Vacuum Workstation (Qiagen). Purified PCR products were mixed with annealing buffer containing the sequencing primer. After the annealing reaction, the plate was loaded into the PyroMark Q96 MD instrument (Qiagen). PyroMark-CpG software automatically generated a dispensation order of deoxynucleoside triphosphates and controlled dispensations based on the sequence to analyze. Controls were included in the dispensation order to check the performance of the reaction. All runs also included a no-template control. To quantify the percentage of DNA CpG methylated, the data were analyzed with the PyroMark software (Qiagen, Germantown, MD, USA). The methylation level was targeted to the region between −427 and −187 in proximal *pparγ* promoter. Specific primers were designed with the Pyro-Mark assay design software (v.2.0; Qiagen, Germantown, MD, USA), which automatically generated primer sets that included both PCR and sequencing primers, based on selected target sequences. One of the primers was biotinylated to enable immobilization to streptavidin-coated beads. The primer sequences were as follows: (forward) 5′-GATGTGTGATTAGGAGTTTTAATTAAAG-3′, (reverse) Biotin-ATCTCTACTCTAATAATTCCAACTA-3′, (sequencing) 5′-CTATTTGGTAAGATTTGGTATATTATAA-3′.

### 2.10. Immunoblotting and Protein Level Determination

Proteins isolated from hepatocytes and liver tissues were analyzed by Western blot as previously described [24]. The following primary antibodies were applied: PPARγ, VLDLR, CD36, DNMT1, DNMT3a, DNMT3b, and β-actin. The specificity and reproducibility of these antibodies were validated prior to this study [15,24]. Band intensity for each protein was analyzed by densitometry (ImageJ version 1.37), and corrections were made using β-actin intensity reading [15].

### 2.11. Gene Expression and QPCR

RNA extraction and synthesis of complementary DNA was performed as previously described [15]. Quantitative polymerase chain reaction (qPCR) was performed using SYBR Green Supermix with iTaqDNA polymerase on the IQ5 thermocycler, and specifically designed and optimized oligonucleotides [15,24]. The sequences of the oligonucleotides used for selected genes were as follows: *pparγ* forward: 5′-CGGTTTCAGAAGTGCCTTG-3′; reverse: 5′-GGTTCAGCTGGTCGATATCAC-3, *vldlr* forward: 5′-GATGATGACGCAGACTGTTC-3′; reverse: 5′-CACTGGATCTCACTGGTAGG-3′, *cd36* forward: 5′-CTGTTATTGGTGCAGTCCTGGC-3′; reverse: 5′-TATGTGGTGCAGCTGCTACAGC-3′, *dgat2* forward: 5′-AGTGGCAATGCTATCATCATCGT-3′; reverse: 5′-AAGGAATAAGTGGGAACCAGATCA-3′, *fatp* forward: 5′-TGGACCCAAAGTGGTCCGCA-3′; reverse: 5′-AGTTCAGTCACGGACTTTAT-3′. *fabp* forward: 5′-GGGGGTGTCAGAAATCGTG-3′; reverse: 5′ CAGCTTGACGACTGCCTTG-3′, *dnmt1* forward: 5′-ATGAGAGGGAGGAGAAGAGAC-3′; reverse: 5′- TGCTGCTGGTACTTCAGGTTAG-3′, *dnmt3a* forward: 5′-CATGAACAGGCCTTTGGCA-3′; reverse: 5′-TCTTGCAGCTCCAGCTTATC-3′, *dnmt3b* forward: 5′-CCAAAAGGAGGCCCATTAGAG-3′; reverse: 5′-GTACCCCGTTGCAATTCCAT-3′, and *18S* forward: 5′-ACAGGATTGACAGATTGA-3′; reverse:5′-TATCGGAATTAACCAGACA-3′. Data of qPCR were obtained as CT values, defined as the threshold cycle of PCR where products amplify exponentially. Difference in the CT values (ΔCT) was derived from the specific gene tested and CT of the control gene 18S according to the equation 2^[CT18S^-^CTtarget gene]^, as described previously [22].

### 2.12. Statistical Analysis

Averaged values are presented as means ± SEM. Statistical analysis was assessed with unpaired Student’s t test for two group comparison, and two-way analysis of variance (ANOVA) followed by Tukey’s post-hoc test for multiple groups. Results were considered statistically significant if *p* was less than 0.05. All *p* values were two-sided. GraphPad Prism 6 (GraphPad Software, San Diego, CA, USA) was used for all analyses.

## 3. Results

### 3.1. Blood Parameter Analysis

As expected, body weight and gonadal fat and liver weights were strongly increased in HFD-fed mice (+65%, +62%, and +63%, respectively, compared to LFD) (Table 1). In addition, HFD feeding increased the concentrations of plasma TG (+63%), total cholesterol (+29%), free fatty acids (+47%), glucose (+23%), and insulin (+320%). Moreover, HFD enhanced plasma concentrations of inflammation parameters IL−6 (+123%) and TNF-α (+90%), as well as liver enzymes, alanine aminotransferase (+95%), and aspartate aminotransferase (+48%).

### 3.2. High-Fat Diet Was Associated with Hepatic Steatosis and Inflammation

**Figure 1:**
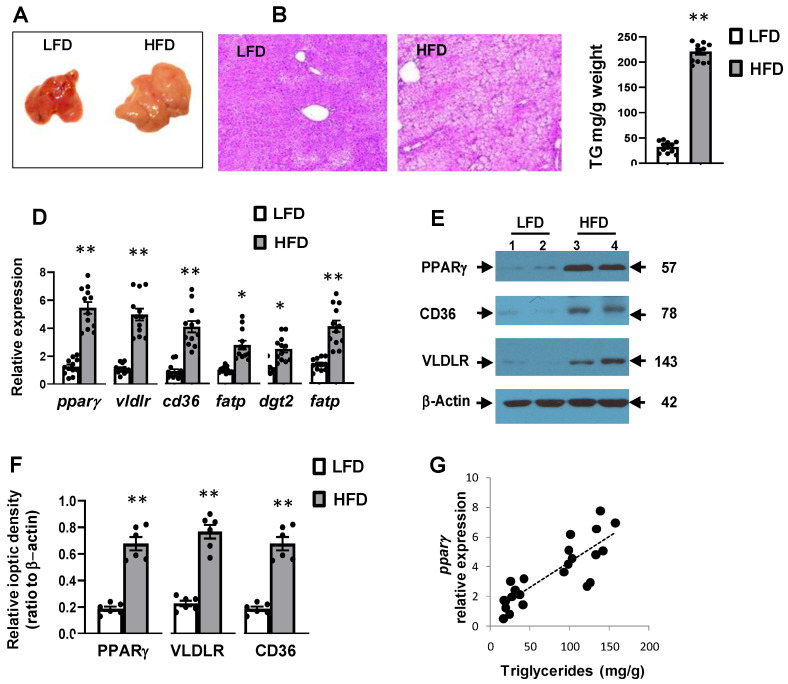
Effects of high-fat diet on hepatic lipids and gene expression. (**A**) Representative photos of gross liver and (**B**) microscopic hematoxilin and eosin (H&E) stained sections. (**C**) Hepatic TG content (*n* = 12), (**D**) levels of mRNA (*n* = 12), (**E**) representative blots, and (**F**) means of optic density of protein bands (*n* = 6). (**G**) Association between hepatic triglyceride content and *pparγ* expression. Mice were fed low fat diet (LFD) and high-fat diet (HFD) for 12 weeks. Hepatic triglycerides were measured by enzymatic assay. Results are presented as Mean ± SEM. Statistical difference was assessed with unpaired *t*-test, differently from LFD with ** *p* < 0.01. The abundance of mRNA was investigated with qPCR, differently from LFD with ** *p* < 0.01 and * *p* < 0.05. Protein levels were examined by Western blotting and data of optical density were arbitrary units and were obtained after scans of bands, as described in Methods. Different from LFD, ** *p* < 0.01.

The liver of HFD-fed mice exhibited the features of fatty liver with enlargement size and pale color (Figure 1A). This was also evident in microscopic sections stained with H&E showing the presence of fine and coarse fat droplets (Figure 1B), and direct measurement of hepatic TG content, which was about 6-fold higher in HFD than in LFD (Figure 1C).

### 3.3. High-Fat Diet Induced Hepatic Expression of Pparγ and Its Target Genes

To assess the molecular basis that could be linked to hepatic steatosis, we examined the expression of several genes involved in lipid metabolism. While the abundance of hepatic *pparγ* mRNA was very low in LFD mice, it was strongly enhanced by HFD feeding (Figure 1D). Similarly, HFD feeding increased the expression of the *vldlr*, *cd36*, *fatp*, *dgat2*, and *fabp* genes known to be involved in cellular lipid uptake and storage (Figure 1D). In agreement with this, the protein levels of PPARγ, VLDLR, and CD36, which were very low in LFD mice, were markedly enhanced in the liver of HFD-fed mice (Figure 1E,F). There was a positive correlation between *pparγ* mRNA abundance and hepatic TG content (r = 0.59, *p* < 0.05) (Figure 1F). Similarly, mRNA abundance of *vldlr* and *cd36* was positively associated with hepatic TG (r = 0.53 and 0.61, respectively, *p* < 0.05).

### 3.4. High-Fat Diet Increased Fatty Acid and Very Low Density Lipoprotein Uptake in Hepatocytes

**Figure 2:**
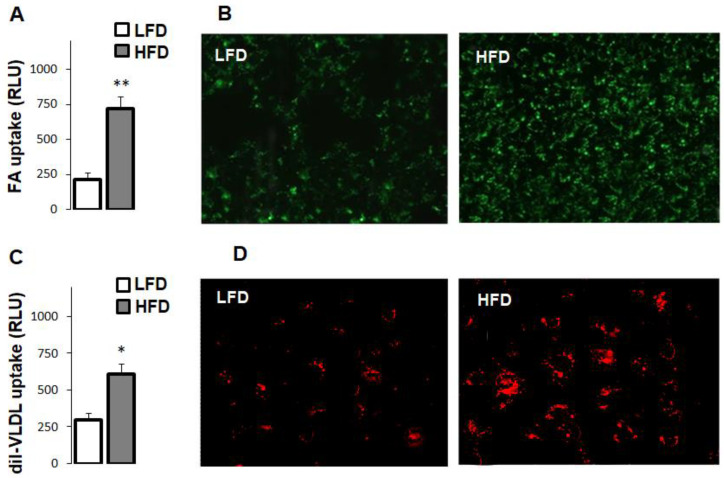
Effects of high-fat diet on fatty acid and VLDL uptake. Uptake of fatty acids (**A**) and VLDL (**C**) were measured in isolated hepatocytes (6 mice per group) using Bodipy C16 and diI-VLDL, as described in Methods. Statistical difference was assessed with unpaired *t*-test with ** *p* < 0.01 and * *p* < 0.05. Microscopic images of hepatocytes incubated with Bodipy C16 (**B**) and diI VLDL (**D**) showed intracellular fluorescence.

To further assess the effects of high-fat diet in the liver, we measured fatty acid uptake in hepatocytes using a synthetic fatty acid analogue. Compared to LFD, fatty acid uptake was about three-fold higher in the hepatocytes of HFD-fed mice (Figure 2A). This difference was also evident in microscopic images showing markedly stronger fluorescence intensity in the hepatocytes of HFD-fed mice (Figure 2B). Similarly, HFD increased hepatocyte uptake of VLDL, as shown by the direct reading of intracellular VLDL fluorescence (Figure 2C) and microscopic images (Figure 2D).

### 3.5. High-Fat Diet Altered Hepatic Global DNA Methylation

**Figure 3:**
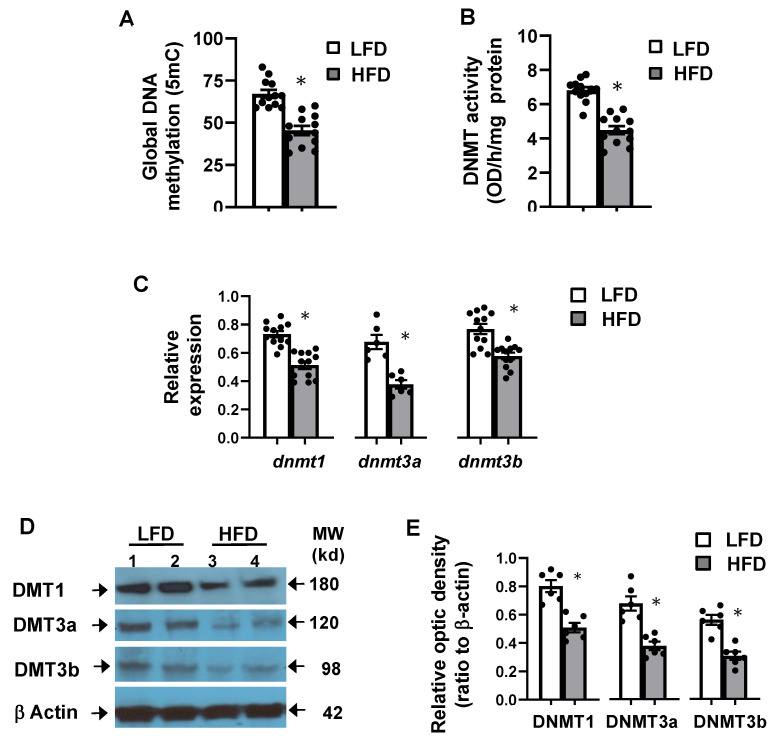
High-fat diet altered DNA methylation, and DNA methyltransferases (DNMT) activity and expression. (**A**) Global DNA methylation (*n* = 12) and (**B**) activity (*n* = 12) and (**C**) mRNA abundance of DMNT (*n* = 12). (**D**) Representative protein blots and (**E**) optic density of protein bands (*n* = 6) of hepatic DNMT of mice fed LFD and HFD diets. Statistical difference was assessed with unpaired *t*-test with * *p* < 0.05.

To gain further insight on the molecular changes associated with high-fat diet, we assessed hepatic DNA methylation. Global DNA methylation analysis (Figure 3A) indicates that HFD diet induced a 25% reduction of hepatic 5mC (*p* < 0.05). Moreover, the activity of DNMT (Figure 3B) was about 31% lower in the liver of HFD-fed mice, and this was associated with a significant reduction of *dnmt* mRNA abundance (Figure 3C) and protein levels (Figure 3D,E). Although protein levels of all DNMT isoforms were altered, DNMT3a and DNMT3b were the most reduced (Figure 3C). These results indicate that hepatic HFD-induced steatosis was associated with DNA demethylation and reduction of DNMT expression and activity.

### 3.6. High-Fat Diet Reduced Pparγ Promoter Methylation

**Figure 4:**
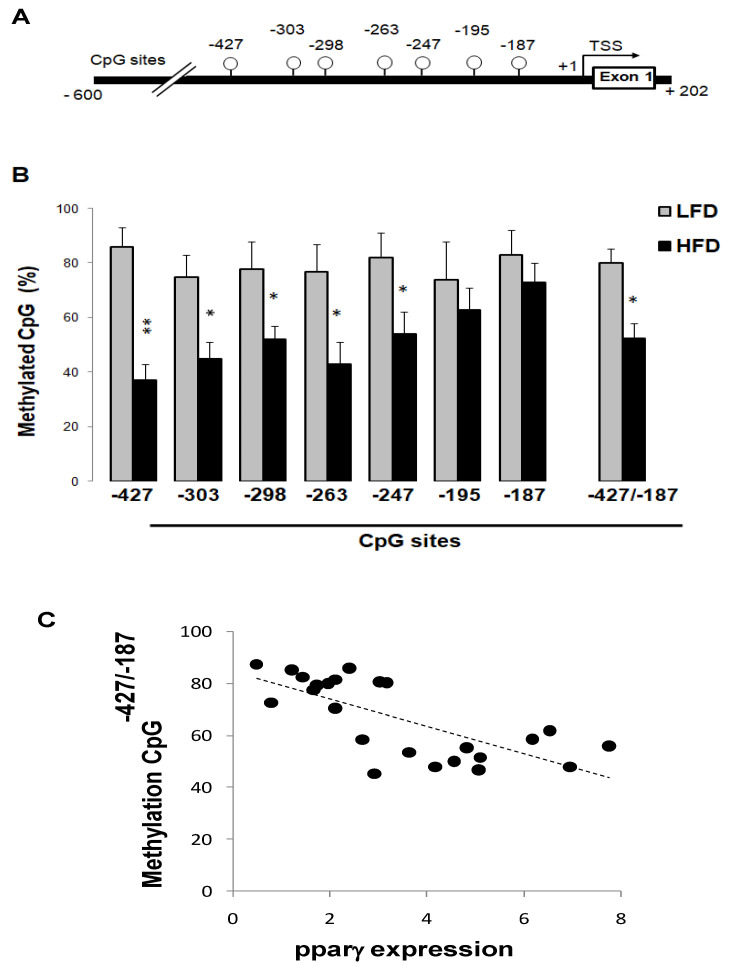
High-fat diet reduced *pparγ* DNA promoter methylation. Representative map of CpG sites in *pparγ* DNA promoter (**A**), methylation level of CpG sites in *pparγ* promoter (**B**),and correlation of *pparγ* mRNA and *pparγ* promoter methylation level (**C**). Hepatic DNA was isolated from mice fed LFD and HFD, and the DNA methylation profile of individual CpG sites in the *pparγ* promoter was assessed with bisulfate pyrosequencing analysis (*n* = 8), as described in Methods. Different from LFD, ** p < 0.01 and * *p* < 0.05.

A preliminary online search revealed that the 5′-untranslated region of the *pparγ* gene is enriched in CpG sites (Figure 4A), which raised the possibility of epigenetic regulation through DNA methylation. We therefore assessed the methylation status of seven CpG sites using pyroseqencing procedures (Figure 4B). The level of methylation differed between LFD and HFD diets, but this difference varied according to CpG site location. In LFD mice, the level of methylation of the tested CpG sites was between 75 and 84%. By contrast, the HFD diet induced marked and significant reductions of the methylation level in CpG^−427^ (−57%), CpG^−303^ (−40%), CpG^−298^ (−34%), CpG^−263^ (−45%), and CpG^−247^ (−35%) sites but did not significantly alter the methylation at CpG^−195^ (−14%) and CpG^−187^ (−5%). When considered all together, the average methylation of CpG^−427/−187^sites was about 38% lower in HFD-fed mice (*p* < 0.05). There was a significant inverse correlation between the level of CpG^−427/−187^methylation and *pparγ* gene expression (r = 0.57, *p* < 0.05) (Figure 4C). Similar inverse correlation was also detected with individual CpG sites, but the statistical significance varied from site to site; significant correlation with the methylation levels of CpG^−427^, CpG^−303^, CpG^−298^, and CpG^−263^ (r = 0.61 to 0.52, *p* < 0.05), but not with CpG^−195^and CpG^−187^. These results suggest that the epigenetic regulatory mechanism is a key player in hepatic *pparγ* gene expression in steatosis conditions.

### 3.7. Palmitic Acid Regulated DNA Methylation and Expression of Pparγ and Its Target Genes in Hepatocytes

**Figure 5:**
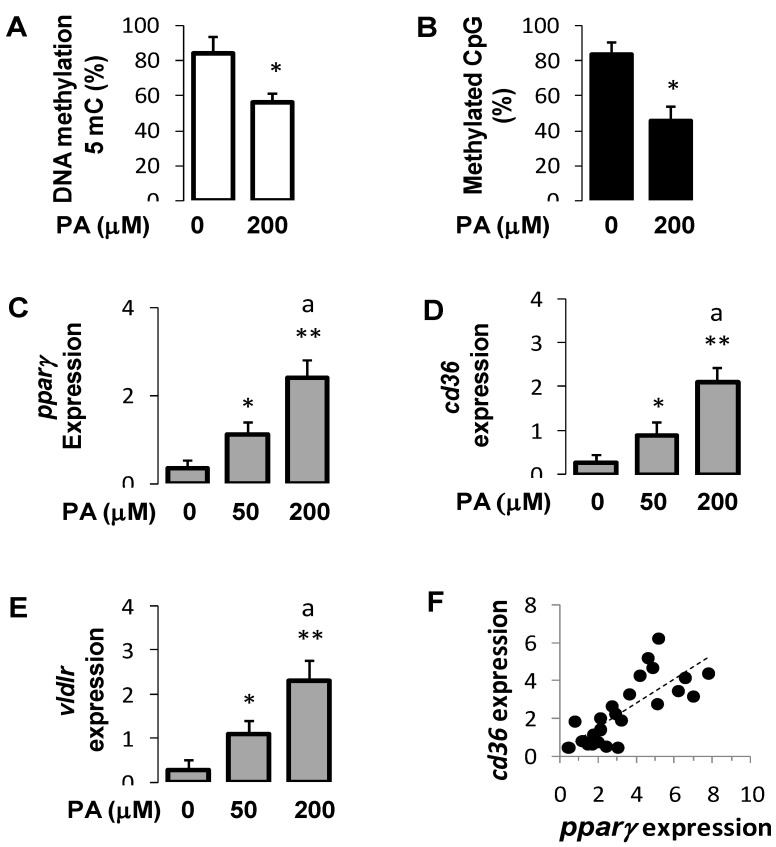
Effects of palmitic acid DNA methylation and gene expression in hepatocytes. Global DNA methylation(**A**); *pparγ* promoter CpG site methylation (**B**); expression of *pparγ* (**C**), *cd36* (**D**), and *vldlr* (**E**); and correlation between *pparγ* and *cd36* expression (**F**). Primary hepatocytes isolated from six mice were incubated with BSA without palmitic acid (control) or with palmitic acid (50 and 200 μM) for 24 h. The levels of mRNA was assessed with qPCR (*n* = 12 per condition). Data were generated from three independent experiments in which each experimental condition was carried out in duplicates. Data are mean ± SEM. Statistical analysis was performed with ANOVA test followed by Tukey’s test. Significance between palmitic treated and control (untreated, PA 0 μM) is represented with asterisks, * *p* < 0.01 and ** *p* < 0.001, and between 50 and 200 μM, ^a^ *p* < 0.05.

To assess the specific effects of fatty acids on *pparγ* gene expression, we challenged hepatocytes with palmitic acid (PA), which was the predominant fatty acid in the HFD diet. Like HFD, chronic overload of PA reduced the global DNA methylation (Figure 5A) and the *pparγ* promoter-specific DNA methylation (Figure 5B). This was associated with a dose-dependent increase in *pparγ* mRNA abundance (Figure 5C) as well as that of *cd36* (Figure 5D) and *vldlr* (Figure 5E). There were positive associations between the expression of *pparγ* and *cd36* (*p* < 0.61, *p* < 0.05) (Figure 5F) and between *pparγ* and *vldlr* (r = 0.65, *p* < 0.05). These findings indicate that PA exerted epigenetic modifications similar to those induced by HFD feeding.

### 3.8. Pharmacological Inhibition of DNMT Increased PparγExpression in Hepatocytes

**Figure 6:**
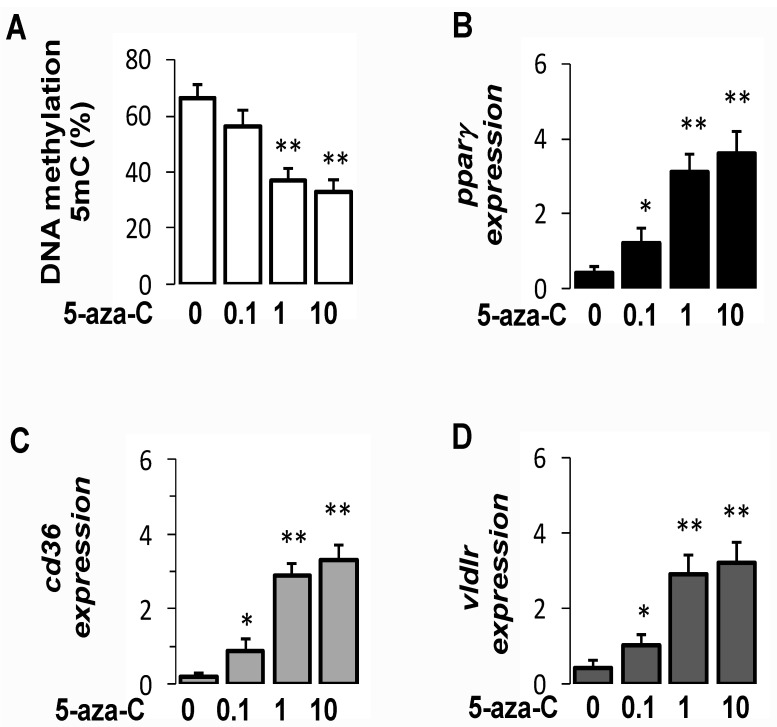
Inhibition of DNA methylation altered the expression of *pparγ* and its target genes. The effects of 5′-aza, 2′ deoxycytidine (5-aza-C) on global DNA methylation (**A**), and expression of *pparγ* (**B**), *cd36* (**C**), and *vldlr* (**D**) in hepatocytes treated with 5-aza-C. Primary hepatocytes were incubated in medium without 5-aza-C (control) or with increasing concentrations of 5-aza-C for 48 h. Expression of selected genes was assessed with qPCR, as described in Methods. Experiments were conducted in duplicate, and results were generated from three experiments. Results are presented as mean ± SEM (*n* = 12 per experimental condition). Significance was assessed with ANOVA test followed with Tukey’s test. Significance compared to control (without 5-aza-C) is indicated with an asterisk with * *p* < 0.01 and ** *p* < 0.001.

To further investigate the links between DNA methylation and *pparγ* expression, we challenged hepatocytes with 5′-aza, 2′ deoxycytidine (5-aza-C), a strong specific inhibitor of DNMT [26]. As expected, 5-aza-C significantly reduced global DNA methylation, as indicated by a lower proportion of 5mC global (Figure 6A). In addition, DNMT inhibition was associated with higher expression of *pparγ* (Figure 6B), *cd36* (Figure 6C), and *vldlr* (Figure 6D) in hepatocytes. This suggests that DNMT-mediated DNA methylation was directly responsible, at least in part, for low expression of *pparγ* and its target genes in hepatocytes.

## 4. Discussion

Our findings demonstrate that both HFD and palmitic acid alter global and *pparγ*-promoter DNA methylation, leading to strong induction of the expression of PPARγ, CD36, and VLDLR and enhanced fat accumulation in the liver, an initiation step in the development of NAFLD.

While it is known that epigenetic modifications are important mediators of diet-induced gene expression, the mechanisms governing region-specific DNA methylation and their role in NAFLD pathogenesis are poorly understood, specifically in the context of nutriepigenomics [29]. In the present study, high-fat diet feeding led to the expected effects of increased body weight, marked expansion of adipose tissue and NAFLD. In addition, high-fat diet-induced hepatic steatosis was associated with significant alteration of global and specific *pparγ* promoter DNA hypomethylation concomitantly with a reduction of DNMT activity and expression. Furthermore, the in vitro investigation provided novel findings in that hepatocytes treated with palmitic acid alone exhibited comparable changes in *pparγ* promoter demethylation than those seen in the liver of HFD-fed mice. These results provide a strong argument that dietary palmitic acid contributes, at least in part, to the changes of the DNA methylation profile induced by HFD, and most likely plays an important role in the induction of hepatic PPARγ expression. These findings are in line with previous investigations reporting that fatty acid composition in blood is associated with the level of DNA methylation in blood cells [13] and that dietary fatty acids are correlated with the methylation level of several genes in the liver [30] and other organs [31,32].

Another important finding of this study is that pharmacological inhibition of DNMT in hepatocytes led to the upregulation of PPARγ and its target genes. These findings highlight the significance of epigenetic regulation in hepatic gene modulation. In line with our results, previous investigations have reported that 5-aza-C induced LDLR expression in a dose-dependent manner, and enhanced cholesterol content in hepatocytes [26]. Of note, LDLR and VLDLR belong to the same family of receptors and they may also share comparable mechanisms of regulation. In a comparable manner, palmitic acid and HFD blunted DNMT expression and reduced *pparγ* promoter DNA methylation while increasing the expression of PPARγ and its target genes. These results indicate that modulation of DNMT could be an important factor that mediates nutrients’ effects on PPARγ expression. In support of this mechanism of action, high-fat diet reduces DNMT activity/expression in the liver and peripheral tissues and enhances the expression of several genes, including fatty acid synthase [33,34]. More importantly, while *pparγ* expression was suppressed by DNMT over-expression [35], it was enhanced in macrophages treated with 5-aza-C [36]. Similarly, silencing DNMT isoform DNMT3b with small interference RNA (siRNA) in myocytes enhanced global and gene-specific DNA methylation, leading to the induction of several genes including *TNFα*, peroxisome proliferator-activated receptor γ coactivator-1 α (*PGC-1α*) [37]. It is thus conceivable that palmitic acid regulates *pparγ* gene expression through epigenetic regulation involving the suppression of DNMT, thereby increasing promoter DNA hypomethylation. A similar mechanism of action has been proposed to explain the effects of n-6 PUFA and n3 PUFA on *TNF-α* promoter DNA methylation [8,38,39]. Beside the modulation of DNMT, other possible mechanisms could involve the interaction of palmitic acid with proteins needed for DNA methylation, such as methyl CpG binding protein 2 (MeCP2) [8] and ten-eleven translocation (TET) protein [40]. Interestingly, the amount of S-adenosylmethionine, a metabolite of methionine and a principal methyl donor required for DNMT-meditated DNA methylation, is altered by a high-fat diet, an effect that could also affect DNMT activity [41].

One of the main functions of PPARγ is promoting the storage of excess lipids through the activation of a network of target genes [15]. In the present study, up-regulation of PPARγ expression, whether with high-fat diet or palmitic acid overloaded, was associated with the induction of VLDLR and CD36 expression, and increased lipid uptake and accumulation. This is consistent with the fact that these genes are under the control of PPARγ, by which it promotes lipid uptake and storage [15]. It is known that CD36 facilitates fatty acid uptake in several cell types, and its level of expression regulates the size of intracellular lipid content, as has been demonstrated by knockdown and over-expression manipulations [16]. Likewise, VLDLR plays a crucial role in TG-rich lipoprotein catabolism, directly as a receptor of remnant particle or as a facilitator of lipoprotein lipase (LPL) action and TG hydrolysis [15]. In previous studies, we have demonstrated that activation of PPARγ increases VLDLR expression and enhances remnant particle uptake and intracellular lipid accumulation [15,16]. In this study, the expression level of hepatic PPARγ is positively associated with the expression of VLDLR and CD36, as well as the amount of hepatic TG. In addition, induction of PPARγ expression in hepatocytes treated with palmitic acid and 5-aza-C goes together with elevated expression of VLDLR and CD36, and higher uptake of VLDL and FFA. Therefore, it is likely possible that both VLDLR-mediated remnant uptake and CD36-mediated fatty acid uptake contribute to increase hepatic lipid accumulation and NAFLD pathogenesis. This hypothesis is rendered more plausible by the fact that HFD feeding increased the availability of FFAs and TG-rich lipoproteins, both of which were markedly increased in the blood.

It has been shown that regulation of PPARγ expression is tissue-specific [42]. While upregulation of PPARγ expression is linked to obesity, activation of this receptor has also been shown to enhance insulin sensitivity and accelerate blood triglyceride clearance, hence preventing insulin resistance and hypertriglyceridemia [42]. In this sense, induction of adipose tissue PPARγ expression could be considered as a beneficial response designed to accelerate blood lipid clearance, directed primary towards safe storage in adipose tissue. With chronic HFD feeding, upregulation of PPARγ expression in adipose tissue may not be sufficient to accommodate a continued overload of dietary lipids. In this situation, induction of hepatic PPARγ expression could be seen as a second mechanism of regulation of triglyceride homeostasis. Although contributing to hepatic steatosis, this action could protect other tissues from triglyceride accumulation and insulin resistance [42].

**Figure 7:**
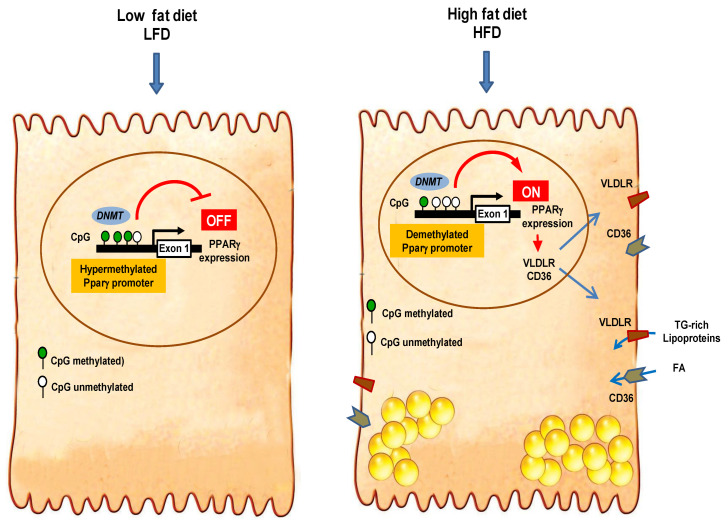
Proposed mechanism of action of high-fat diet and palmitic acid underlying modification of *pparγ* promoter DNA methylation and the expression of *pparγ* and its target genes in hepatocytes. In low fat diet (LFD), most CpG sites in *pparγ* DNA promoter are hypermethylated, and the expression of *pparγ* and its target genes *vldlr* and *cd36* is virtually absent. High-fat diet and palmitic acid overload reduces DNMT activity, leading to the demethylayion of most CpG sites of the *pparγ* DNA promoter and upregulation of *pparγ* expression, which in turn induces the expression of *vldlr* and *cd36*, increasing the uptake of fatty acids and triglycerides in hepatocytes.

Although the importance of CD36 and VLDLR in lipid uptake has been well documented in adipose tissue, it is deemed to be irrelevant in the liver due to low expression in healthy conditions and consumption of a low-fat diet. Our study provides strong evidence that both receptors become important players in hepatic lipid uptake in HFD feeding. As such, they mediate the lipogenic function of PPARγ in the liver in a similar fashion to that in adipose tissue. In preadipocytes, the precursor cell of adipocytes, the expression of PPARγ is almost undetected and is the same as that of CD36 and VLDLR. During the differentiation of preadipocytes, PPARγ is first upregulated followed by the induction of CD36 and VLDLR, leading to increased uptake of FA and TG-rich lipoproteins and accumulation of neutral lipids [15]. Therefore, we can speculate that a comparable mechanism might occur in the liver in response to the consumption of a HFD, which expands lipid concentrations in blood beyond the storage capacity of adipose tissue (Figure 7). Excess fatty acids reaching hepatocytes might first induce *pparγ* gene and then its target genes *cd36* and *vldlr*, leading to increased lipid uptake and storage. By promoting hypomethylation of hepatic *pparγ* DNA promoter, palmitic acid seems to play a crucial role in the epigenetic regulation of this chain of events, leading to hepatic steatosis.

This study focused on the role of palmitic acid and a saturated fat-rich diet on epigenetic regulation of PPARγ and hepatic lipid accumulation. The question whether similar mechanisms of action also apply to polyunsaturated (PUFAs) and monounsaturated fatty acids (MUFAs) is still to be elucidated. It is known that saturated fatty acids (SFA), MUFAs and PUFAs regulate differentially gene expression, inflammation and lipid metabolism [39,43]. The Western-style dietary pattern is typically characterized by a high intake of SFAs with low amounts of n-3 PUFAs and MUFAs [2]. In addition to being a risk for NAFLD, high amounts of SFA consumption are associated with decreased fatty acid oxidation and increased intracellular lipid accumulation [2]. By contrast, diets rich in PUFA, mostly n-3 PUFAs, increase fatty acid oxidation and could be associated with a reduction of intra-hepatic lipid contents [44,45]. Therefore, it is possible that replacing SFAs with MUFAs or n-3 PUFAs could reduce NAFLD; however, additional studies are needed to assess this hypothesis and to investigate the effects of MUFAs and PUFAs on epigenetic regulation on PPARγ and hepatic and NAFLD pathogenesis.

In summary, this study revealed that consumption of Western-type high-saturated fat diet induces hepatic steatosis in association with the reduction of DNMT activity, hypomethylation of *pparγ* DNA promoter, and upregulation of PPARγ and its target genes *vldlr* and *cd36*, leading to increased lipid uptake. The fact that palmitic acid overload engenders comparable modifications in hepatocytes suggests that epigenetic modification induced by dietary fatty acids is an important regulator of hepatic PPARγ and might play an important role in diet-induced NAFLD pathogenesis. The results of this study also provide strong evidence that epigenetic regulation, through *pparγ* DNA methylation, is an important mechanism of regulation of hepatic PPARγ expression, and hence intra-hepatic lipid accumulation.

## Figures and Tables

**Table 1:** Characteristics and plasma parameters for mice fed low-fat diet (LFD) and high-fat diet (HFD).

Characteristics	LFD(*n* = 16)	HFD(*n* = 16)
Body weight final (g)	26 ± 4	43 ± 7 **
Liver weight (g)	2.4 ± 0.3	3.9 ± 0.3 ***
Gonadal fat weight (g)	1.9 ± 0.1	3.1 ± 0.2 ***
TG (mg/dL)	82 ± 5	134 ± 10 **
Cholesterol (mg/dL)	87.6 ± 7.6	113.4 ± 10.2 **
FFAs (mmol/L)	0.74 ± 0.10	1.09 ± 0.14 **
Glucose (mg/dL)	86 ± 6	106 ± 8 **
Insulin (ng/mL)	2.4 ± 0.6	10.1 ± 0.9 ***
IL-6 (ng/mL)	13 ± 3	29 ± 6 **
TNF-α (ng/mL)	10 ± 4	19 ± 6 *
ALT(mU/mL)	7.3 ± 0.9	19.3 ± 1.5 *
AST (mU/mL)	15.6 ± 3.1	23.1 ± 5.2 *

Data are expressed as mean ± SEM. Statistical significance was assessed with unpaired Student’s *t*-test, with * *p* < 0.05, ** *p* < 0.01, and *** *p* < 0.001. Abbreviations: ALT, Alanine aminotransferase; AST, Aspartate aminotransferase; FFA, Free fatty acids; IL-6, Interleukine-6; TG, Triglycerides.

## Data Availability

No new data outside those presented in this study were created or analyzed. Data sharing is not applicable to this article.

## Supplementary Materials

The following are available online at https://www.mdpi.com/article/10.3390/cells10061355/s1. Table S1: Composition of low fat diets (LFD) and high-fat diets (HFD).
