# Supplementary material for: Epigenetic Regulation of Peroxisome Proliferator-Activated Receptor Gamma Mediates High-Fat Diet-Induced Non-Alcoholic Fatty Liver Disease
Source: Cells. 2021 May 31;10(6):1355. doi: 10.3390/cells10061355 (PMC8229510; doi:10.3390/cells10061355)
Supplement: Supplementary file 1 [file cells-10-01355-s001.zip › cells-1216945-supplementary.pdf]

Supplementary Materials

Table S1: Composition of low fat (LFD) and high fat (HFD) diets

The top part of the table shows the percent composition of macronutrients and the lower part of shows detailed ingredients' composition.

Diets	Low fat diet (LFD)		High fat diet (HFD)	
Product number	D13052302		D13052306	
%	g (%)	Kcal (%)	g (%)	Kcal (%)
Protein	19.2	20.0	26.2	20.0
Carbohydrate	67.3	70.0	26.3	20.1
Fat	4.3	10.0	34.9	59.9
Total		100		100
Kcal/gm		3.85		5.24
Ingredients	g	kcal	g	kcal
Casein	200	800	200	800
L-Cystine	3	12	3	12
Corn Starch	506.2	2024.8	0	0
Maltodextrin 10	125	500	125	500
Sucrose	68.8	275.2	68.8	275.2
Cellulose	50	0	50	0
Soybean oil	25	225	25	225
Cocoa butter	20	180	20	180
Palm oil	0	0	225	2025
Mineral Mix S10021	10	0	10	0
Dicalcium Phosphate	13	0	13	0
Calcium carbonate	5.5	0	5.5	0
Potassium Citrate	16.5	0	16.5	0
Vitamin Mix V10001	10	40	10	40
Choline Bitartrate	2	0	2	0
Red Dye, FD&C #40	0	0	0.05	0
Blue Dye, FD&C #5	0.05	0		0
Total	1055.05	4057	773.85	4057.2
